# On stability analysis for generalized Minty variational-hemivariational inequality in reflexive Banach spaces

**DOI:** 10.1186/s13660-018-1890-9

**Published:** 2018-10-30

**Authors:** Lu-Chuan Ceng, Ravi P. Agarwal, Jen-Chih Yao, Yonghong Yao

**Affiliations:** 1 Department of Mathematics, Shanghai Normal University, Shanghai, China; 2 Department of Mathematics, Texas A&M University, Kingsville, USA; 3 Florida Institute of Technology, Melbourne, USA; 4 Center for General Education, China Medical University, Taichung, Taiwan; 5 Department of Mathematics, Tianjin Polytechnic University, Tianjin, China

**Keywords:** Generalized variational-hemivariational inequality, Stability, Clarke’s generalized directional derivative, Pseudomonotone mapping, Reflexive Banach space

## Abstract

The stability for a class of generalized Minty variational-hemivariational inequalities has been considered in reflexive Banach spaces. We demonstrate the equivalent characterizations of the generalized Minty variational-hemivariational inequality. A stability result is presented for the generalized Minty variational-hemivariational inequality with $(f,J)$-pseudomonotone mapping.

## Introduction

Let *X* be a real Banach space with its dual $X^{*}$. Let $K\subset X$ be a nonempty, closed, and convex set. Let $F:K\to2^{X^{*}}$ be a set-valued mapping. Let $A:K\to X^{*}$ be a single-valued mapping. Let $f:K\subset X\to\mathbf{R}\cup\{+\infty\}$ be a proper, convex, and lower semicontinuous functional. Let $J:X\to\mathbf{R}$ be a locally Lipschitz functional. We use $J^{\circ}(\cdot,\cdot)$ to denote Clarke’s generalized directional derivative of *J*. Recall that the variational-hemivariational inequality [1] can mathematically be formulated as the problem of finding a point $u\in K$ such that

1.1 $$ \operatorname{VHVI}(A,J,K): \langle Au,v-u\rangle+J^{\circ}(u,v-u)+f(v)-f(u) \geq0,\quad \forall v\in K. $$

In particular, if $J=0$, then the $\operatorname{VHVI}(A,J,K)$ reduces to the following mixed variational inequality of finding $u\in K$ such that

1.2 $$ \operatorname{MVI}(A,K): \langle Au,v-u\rangle+f(v)-f(u)\geq0,\quad \forall v\in K. $$

MVI has been studied extensively in the literature, see, for instance, [2–6].

Under some suitable conditions, (1.2) is equivalent to the following Minty mixed variational inequality [7–15] which is to find $u\in K$ such that

1.3 $$ \operatorname{MMVI}(A,K): \langle Av,v-u\rangle+f(v)-f(u)\geq0, \quad\forall v\in K. $$

In the present paper, we consider the following generalized Minty variational-hemivariational inequality of finding $u\in K$ such that

1.4 $$ \operatorname{GMVHVI}(F,J,K): \sup_{v^{*}\in F(v)}\bigl\langle v^{*},u-v\bigr\rangle +J^{\circ}(v,u-v)+f(u)-f(v)\leq0, \quad\forall v\in K. $$

Special cases: (i) If $J=0$, then (1.4) reduces to the following generalized Minty mixed variational inequality of finding $u\in K$ such that

1.5 $$ \operatorname{GMMVI}(F,K): \sup_{v^{*}\in F(v)}\bigl\langle v^{*},u-v \bigr\rangle +f(u)-f(v)\leq 0, \quad\forall v\in K. $$

(ii) If $F=A$ and $f=0$, then (1.5) reduces to the following classical Minty variational inequality of finding $u\in K$ such that

1.6 $$ \operatorname{MVI}(A,K): \langle Av,u-v\rangle\leq0,\quad \forall v\in K. $$

Let $(Z_{1},d_{1})$ and $(Z_{2},d_{2})$ be two metric spaces. $L:Z_{1}\to2^{X}$ be a set-valued mapping with nonempty, closed, and convex values. Let $F:X\times Z_{2}\to2^{X^{*}}$ be a set-valued mapping. Let $f:X\to\mathbf {R}\cup\{+\infty\}$ be a proper, convex, and lower semicontinuous functional. Next, we consider the following parameter generalized Minty variational-hemivariational inequality which is to find $x\in L(u)$ such that

1.7 $$\begin{aligned} & \operatorname{GMVHVI}\bigl(F(\cdot,v),J,L(u)\bigr): \sup _{y^{*}\in F(y,v)}\bigl\langle y^{*},x-y\bigr\rangle +J^{\circ}(y,x-y)+f(x)-f(y) \leq0, \\ &\quad \quad \forall y\in L(u). \end{aligned}$$

In particular, if $J=0$, then (1.7) reduces to the following parameter generalized Minty mixed variational inequality: find $x\in K$ such that

1.8 $$ \operatorname{GMMVI}\bigl(F(\cdot,v),L(u)\bigr): \sup_{y^{*}\in F(y,v)} \bigl\langle y^{*},x-y\bigr\rangle +f(x)-f(y)\leq0, \quad\forall y\in L(u). $$

It is well known that the variational inequality theory has wide applications in finance, economics, transportation, optimization, operations research, and engineering sciences, see [16–25]. In 2010, Zhong and Huang [19] studied the stability of solution sets for the generalized Minty mixed variational inequality in reflexive Banach spaces.

Inspired and motivated by the above work of Zhong and Huang [19], we investigate the stability of solution sets for the generalized Minty variational-hemivariational inequality in reflexive Banach spaces. We first present several equivalent characterizations for the generalized Minty variational-hemivariational inequality. Consequently, we show the stability of a solution set for the generalized Minty variational-hemivariational inequality with $(f,J)$-pseudomonotone mapping in reflexive Banach spaces. As an application, we give the stability result for a generalized variational-hemivariational inequality. The results presented in this paper extend the corresponding results of Zhong and Huang [19] from the generalized mixed variational inequalities to the generalized variational-hemivariational inequalities.

## Preliminaries

Let *X* be a real reflexive Banach space. Let $J:X\to\mathbf{R}$ be a locally Lipschitz function on *X*. Clarke’s generalized directional derivative of *J* at *x* in the direction *y*, denoted by $J^{\circ}(x,y)$, is defined by

$$ J^{\circ}(x,y)=\limsup_{z\to x \lambda\downarrow0}\frac{J(z+\lambda y)-J(z)}{\lambda}. $$

Let $f:X\to\mathbf{R}\cup\{+\infty\}$ be a proper, convex, and lower semicontinuous function. Denote by $\partial f:X\to2^{X^{*}}$ and $\overline{\partial}J:X\to2^{X^{*}}$ the subgradient of *f* and Clarke’s generalized gradient of *J* (see [26]), respectively. That is,

$$ \partial f(x)=\bigl\{ z\in X^{*}:f(y)-f(x)\geq\langle z,y-x\rangle, \forall y\in X \bigr\} $$

and

$$ \overline{\partial}J(x)=\bigl\{ u\in X^{*}:J^{\circ}(x,y)\geq\langle u,y \rangle, \forall y\in X\bigr\} . $$

It is known that $\overline{\partial}J(x)=\partial(J^{\circ}(x,\cdot ))(0)$, see [27].

### Proposition 2.1

([1])

*Let* *X* *be a Banach space and* *J* *be a locally Lipschitz functional on X*. *Then we have*:

(i) *The function* $y\mapsto J^{\circ}(x,y)$ *is finite*, *convex*, *positively homogeneous*, *and subadditive*;
(ii) $J^{\circ}(x,y)$ *is upper semicontinuous and is Lipschitz continuous on the second variable*;
(iii) $J^{\circ}(x,-y)=(-J)^{\circ}(x,y)$;
(iv) $\overline{\partial}J(x)$ *is a nonempty*, *convex*, *bounded*, *and weak*^∗^-*compact subset of* $X^{*}$;
(v) *For every* $y\in X$, $J^{\circ}(x,y)=\max\{\langle\xi,y\rangle:\xi\in\overline{\partial}J(x)\}$;
(vi) *The graph of* $\overline{\partial}J(x)$ *is closed in* $X\times (w^{*}-X^{*})$ *topology*, *where* $(w^{*}-X^{*})$ *denotes the space* $X^{*}$ *equipped with weak*^∗^ *topology*, *i*.*e*., *if* $\{x_{n}\}\subset X$ *and* $\{x^{*}_{n}\}\subset X^{*}$ *are sequences such that* $x^{*}_{n}\in\overline{\partial}J(x_{n}), x_{n}\to x$ *in* *X* *and* $x^{*}_{n}\to x^{*}$ *weakly*^∗^ *in* $X^{*}$, *then* $x^{*}\in\overline{\partial}J(x)$.

Let *K* be a nonempty, closed, and convex subset of *X*. Let *Y* be a topological space. We use $\operatorname{barr}(K)$ to denote the barrier cone of *K* which is defined by $\operatorname{barr}(K):=\{x^{*}\in X^{*}:\sup_{x\in K}\langle x^{*},x\rangle<\infty\} $. The recession cone of *K*, denoted by $K_{\infty}$, is defined by $K_{\infty}:=\{d\in X:x_{0}+\mu d\in K, \forall\mu>0, \forall x_{0}\in K\}$. The negative polar cone $K^{-}$ of *K* is defined by $K^{-}:=\{x^{*}\in X^{*}:\langle x^{*},x\rangle\leq0, \forall x\in K\}$. The positive polar cone of *K* is defined as $K^{+}:=\{x^{*}\in X^{*}:\langle x^{*},x\rangle\geq0, \forall x\in K\}$.

Let $f:K\to\mathbf{R}\cup\{+\infty\}$ be a proper, convex, and lower semicontinuous function. The recession function $f_{\infty}$ of *f* is defined by

$$ f_{\infty}(x):=\lim_{t\to+\infty}\frac{f(x_{0}+tx)-f(x_{0})}{t}, $$

where $x_{0}\in\operatorname{Dom}f$.

It is known that

2.1 $$ f(x+y)\leq f(x)+f_{\infty}(y), \quad\forall x\in\operatorname{Dom}f,y\in X, $$

and $f_{\infty}(\cdot)$ satisfies $f_{\infty}(\lambda x)=\lambda f_{\infty}(x)$ for all $x\in X, \lambda \geq0$. According to Proposition 2.5 in [28], we deduce

2.2 $$ f_{\infty}(x)\leq\liminf_{n\to\infty} \frac{f(t_{n}x_{n})}{t_{n}}, $$

where $\{x_{n}\}$ is any sequence in *X* converging weakly to *x* and $t_{n}\to+\infty$.

### Definition 2.2

A set-valued mapping $F:K\subset X\to2^{X^{*}}$ is said to be

(i) upper semicontinuous at $x_{0}\in K$ iff, for any neighborhood $\mathrm{N}(F(x_{0}))$ of $F(x_{0})$, there exists a neighborhood $\mathrm{N}(x_{0})$ of $x_{0}$ such that
  $$ F(x)\subset\mathrm{N}\bigl(F(x_{0})\bigr), \quad\forall x\in\mathrm{N}(x_{0})\cap K; $$
(ii) lower semicontinuous at $x_{0}\in K$ iff, for any $y_{0}\in F(x_{0})$ and any neighborhood $\mathrm{N}(y_{0})$ of $y_{0}$, there exists a neighborhood $\mathrm{N}(x_{0})$ of $x_{0}$ such that
  $$ F(x)\cap\mathrm{N}(y_{0})\neq\emptyset, \quad\forall x\in\mathrm{N}(x_{0})\cap K. $$

*F* is said to be continuous at $x_{0}$ iff it is both upper and lower semicontinuous at $x_{0}$; and *F* is continuous on *K* iff it is both upper and lower semicontinuous at every point of *K*.

### Definition 2.3

The mapping *F* is said to be

(i) monotone on *K* iff, for all $(x,x^{*}),(y,y^{*})$ in the $\operatorname{graph}(F)$,
  $$ \bigl\langle y^{*}-x^{*},y-x\bigr\rangle \geq0; $$
(ii) pseudomonotone on *K* iff, for all $(x,x^{*}),(y,y^{*})$ in the $\operatorname{graph}(F)$,
  $$ \bigl\langle x^{*},y-x\bigr\rangle \geq0 \quad\text{implies that}\quad \bigl\langle y^{*},y-x \bigr\rangle \geq0; $$
(iii) stably pseudomonotone on *K* with respect to a set $U\subset X^{*}$ iff *F* and $F(\cdot)-u$ are pseudomonotone on *K* for every $u\in U$;
(iv) *f*-pseudomonotone on *K* iff, for all $(x,x^{*}),(y,y^{*})$ in the $\operatorname{graph}(F)$,
  $$ \bigl\langle x^{*},y-x\bigr\rangle +f(y)-f(x)\geq0 \quad\Rightarrow\quad\bigl\langle y^{*},x-y \bigr\rangle +f(x)-f(y)\leq0; $$
(v) $(f,J)$-pseudomonotone on *K* iff, for all $(x,x^{*}),(y,y^{*})$ in the $\operatorname{graph}(F)$,
  $$ \bigl\langle x^{*},y-x\bigr\rangle +J^{\circ}(x,y-x)+f(y)-f(x)\geq0 \quad\Rightarrow\quad \bigl\langle y^{*},x-y\bigr\rangle +J^{\circ}(y,x-y)+f(x)-f(y) \leq0. $$

### Definition 2.4

Let $\{A_{n}\}\subset X$ be a sequence. Define

$$ \omega{\mathrm{-}}\limsup_{n\to\infty}A_{n}:=\bigl\{ x\in X: \exists\{n_{k}\}\text{ and }x_{n_{k}}\in A_{n_{k}}\text{ such that }x_{n_{k}}\rightharpoonup x\bigr\} . $$

### Definition 2.5

Let $\psi:X\times X\to\mathbf{R}$ be a function. *ψ* is said to be bi-sequentially weakly lower semicontinuous iff, for any sequences $\{ x_{n}\}$ and $\{y_{n}\}$ with $x_{n}\rightharpoonup x_{0}$ and $y_{n}\rightharpoonup y_{0}$, one has

$$ \psi(x_{0},y_{0})\leq\liminf_{n\to\infty} \psi(x_{n},y_{n}). $$

### Lemma 2.6

([29])

*Let* $K\subset X$ *be a nonempty*, *closed*, *and convex set with* $\operatorname{int}(\operatorname{barr}(K))\neq\emptyset$. *Then there exists no sequence* $\{x_{n}\}\subset K$ *satisfying* $\|x_{n}\|\to\infty$ *and* $\frac{x_{n}}{\|x_{n}\| }\rightharpoonup0$. *If* *K* *is a cone*, *then there exists no sequence* $\{d_{n}\}\subset K$ *with* $\|d_{n}\|=1$ *satisfying* $d_{n}\rightharpoonup0$.

### Lemma 2.7

([30])

*Let* $K\subset X$ *be a nonempty*, *closed*, *and convex set with* $\operatorname{int}(\operatorname{barr}(K))\neq\emptyset$. *Then there exists no sequence* $\{d_{n}\}\subset K_{\infty}$ *with* $\|d_{n}\|=1$ *satisfying* $d_{n}\rightharpoonup0$.

### Lemma 2.8

([30])

*Let* $(Z,d)$ *be a metric space and* $u_{0}\in Z$ *be a given point*. *Let* $L:Z\to2^{X}$ *be a set*-*valued mapping with nonempty values*, *and let* *L* *be upper semicontinuous at* $u_{0}$. *Then there exists a neighborhood* *U* *of* $u_{0}$ *such that* $(L(u))_{\infty}\subset(L(u_{0}))_{\infty}$ *for all* $u\in U$.

### Lemma 2.9

([31])

*Let* *E* *be a Hausdorff topological vector space and* $K\subset E$ *be a nonempty and convex set*. *Let* $G:K\to2^{E}$ *be a set*-*valued mapping satisfying the following conditions*:

(i) *G* *is a KKM mapping*, *i*.*e*., *for every finite subset* *A* *of* *K*, $\operatorname{conv}(A)\subset\bigcup_{x\in A}G(x)$;
(ii) $G(x)$ *is closed in* *E* *for every* $x\in K$;
(iii) $G(x_{0})$ *is compact in* *E* *for some* $x_{0}\in K$.

*Then* $\bigcap_{x\in K}G(x)\neq\emptyset$.

## Boundedness of solution sets

In this section, we introduce several characterizations for the solution set *D* of $\operatorname{GMVHVI}(F,J,K)$.

Let $K\subset X$ be a nonempty, closed, and convex set. Let $F:K\to 2^{X^{*}}$ be a set-valued mapping with nonempty values, $J:X\to\mathbf {R}$ be a locally Lipschitz functional, and $f:K\subset X\to\mathbf{R}$ be a convex and lower semicontinuous function.

### Theorem 3.1

*Suppose* $D\ne\emptyset$. *Then*

$$ D_{\infty}=K_{\infty}\cap\bigl\{ d\in\mathbf{R}^{n}:\bigl\langle y^{*},d\bigr\rangle +J^{\circ}(y,d)+f_{\infty}(d)\leq0, \forall y^{*}\in F(y),y\in K\bigr\} . $$

### Proof

Define a function $\varPhi:X\to\mathbf{R}\cup\{+\infty\}$ by

$$ \varPhi(x):=\sup_{y^{*}\in F(y),y\in K}\frac{\langle y^{*},x-y\rangle+J^{\circ}(y,x-y)+f(x)-f(y)}{\varphi(y,y^{*})}, $$

where $\varphi(y,y^{*}):=\max\{\|y^{*}\|,1\}\max\{\|y\|,1\}\max\{|f(y)|,1\} $. Clearly, *Φ* is a proper, convex, and lower semicontinuous function and so $\varPhi_{\infty}$ is well defined on *X*.

Let $D=\{x\in K:\varPhi(x)\leq0\}$. It is clear that *D* is nonempty. According to formula (2.29) in [32], $\{x\in X:\varPhi(x)\leq r\} _{\infty}=\{d\in X:\varPhi_{\infty}(d)\leq0\}$. Hence

$$ D_{\infty}=\bigl(K\cap\bigl\{ x\in X:\varPhi(x)\leq0\bigr\} \bigr)_{\infty}=K_{\infty}\cap\bigl\{ d\in X:\varPhi _{\infty}(d) \leq0\bigr\} . $$

It remains to prove that

$$ \bigl\{ d\in X:\varPhi_{\infty}(d)\leq0\bigr\} =\bigl\{ d\in X:\bigl\langle y^{*},d\bigr\rangle +J^{\circ}(y,d)+f_{\infty}(d)\leq0, \forall y^{*}\in F(y),y\in K\bigr\} . $$

Let $d\in\{d\in X:\langle y^{*},d\rangle+J^{\circ}(y,d)+f_{\infty}(d)\leq0, \forall y^{*}\in F(y),y\in K\}$ and $x_{0}\in X$ with $\varPhi(x_{0})<\infty$. By virtue of the subadditivity and positive homogeneousness of the function $y\mapsto J^{\circ}(x,y)$, we have

$$ \begin{aligned} &\varPhi(x_{0}+td)-\varPhi(x_{0}) \\ &\quad ={ \sup_{y^{*}\in F(y),y\in K}} \frac{\langle y^{*},x_{0}+td-y\rangle+J^{\circ}(y,x_{0}+td-y)+f(x_{0}+td)-f(y)}{\varphi(y,y^{*})} \\ & \qquad{}-{ \sup_{y^{*}\in F(y),y\in K}}\frac{\langle y^{*},x_{0}-y\rangle+J^{\circ}(y,x_{0}-y)+f(x_{0})-f(y)}{\varphi(y,y^{*})} \\ &\quad\leq{ \sup_{y^{*}\in F(y),y\in K}} \frac{\langle y^{*},x_{0}+td-y\rangle+J^{\circ}(y,td)+J^{\circ}(y,x_{0}-y)+f(x_{0}+td)-f(y)}{\varphi(y,y^{*})} \\ & \qquad{}-{ \sup_{y^{*}\in F(y),y\in K}}\frac{\langle y^{*},x_{0}-y\rangle+J^{\circ}(y,x_{0}-y)+f(x_{0})-f(y)}{\varphi(y,y^{*})} \\ &\quad\leq{ \sup_{y^{*}\in F(y),y\in K}} \frac{\langle y^{*},td\rangle+tJ^{\circ}(y,d)+f(x_{0}+td)-f(x_{0})}{\varphi (y,y^{*})} \quad\text{for any }t>0. \end{aligned} $$

This implies that

$$ \frac{\varPhi(x_{0}+td)-\varPhi(x_{0})}{t}\leq\sup_{y^{*}\in F(y),y\in K} \frac{\langle y^{*},d\rangle+J^{\circ}(y,d)+\frac {f(x_{0}+td)-f(x_{0})}{t}}{\varphi(y,y^{*})}, $$

and so

$$ \varPhi_{\infty}(d)=\lim_{t\to\infty}\frac{\varPhi(x_{0}+td)-\varPhi(x_{0})}{t}\leq0. $$

Therefore,

$$ \bigl\{ d\in X:\bigl\langle y^{*},d\bigr\rangle +J^{\circ}(y,d)+f_{\infty}(d) \leq0, \forall y^{*}\in F(y),y\in K\bigr\} \subset\bigl\{ d\in X: \varPhi_{\infty}(d)\leq0\bigr\} . $$

Conversely, if $d\notin\{d\in X:\langle y^{*},d\rangle+J^{\circ}(y,d)+f_{\infty}(d)\leq0, \forall y^{*}\in F(y),y\in K\}$, then there exist $y\in K$ and $y^{*}\in F(y)$ such that $\langle y^{*},d\rangle+J^{\circ}(y,d)+f_{\infty}(d)>0$. Hence,

$$ \begin{aligned} &\frac{\varPhi(x_{0}+td)-\varPhi(x_{0})}{t}\\ &\quad >\frac{ \frac{\langle y^{*},x_{0}+td-y\rangle+J^{\circ}(y,x_{0}+td-y) +f(x_{0}+td)-f(y)}{\varphi(y,y^{*})}-\varPhi(x_{0})}{t} \\ &\quad\geq\frac{\langle y^{*},x_{0}-y\rangle-J^{\circ}(y,y-x_{0})+f(x_{0})-f(y)-\varphi (y,y^{*})\varPhi(x_{0})}{\varphi(y,y^{*})t} \\ &\qquad{} +\frac{\langle y^{*},d\rangle+J^{\circ}(y,d)}{\varphi(y,y^{*})}+\frac {f(x_{0}+td)-f(x_{0})}{\varphi(y,y^{*})t} \\ &\quad\to\frac{\langle y^{*},d\rangle+J^{\circ}(y,d)+f_{\infty}(d)}{\varphi (y,y^{*})} \quad\text{as } t\to\infty. \end{aligned} $$

This yields that

$$ \varPhi_{\infty}(d)\geq\frac{\langle y^{*},d\rangle+J^{\circ}(y,d)+f_{\infty}(d)}{\varphi(y,y^{*})}>0, $$

and hence the converse inclusion is true. This completes the proof. □

### Corollary 3.2

*Suppose* $D\ne\emptyset$. *Then*

$$ D_{\infty}=K_{\infty}\cap\bigl\{ d\in X:\bigl\langle y^{*},d\bigr\rangle +f_{\infty}(d)\leq0, \forall y^{*}\in F(y),y\in K\bigr\} . $$

### Proof

If $J=0$, then $J^{\circ}=0$. In this case, $\operatorname{GMVHVI}(F,J,K)$ reduces to $\operatorname{GMMVI}(F,K)$. Utilizing Theorem 3.1, we immediately deduce Corollary 3.2. □

### Remark 3.3

It is known that if $J=0$ then Theorem 3.1 reduces to Zhong and Huang’s one [19, Theorem 3.1]. Thus, Theorem 3.1 generalizes and extends Theorem 3.1 in Zhong and Huang [19] from $\operatorname{GMMVI}(F,K)$ to $\operatorname{GMVHVI}(F,J,K)$. If $f=0$ additionally, then $f_{\infty}=0$ and so

$$ D_{\infty}=K_{\infty}\cap\bigl\{ d\in X:\bigl\langle y^{*},d\bigr\rangle \leq0, \forall y^{*}\in F(K)\bigr\} =K_{\infty}\cap F(K)^{-}. $$

Hence, Zhong and Huang’s Theorem 3.1 in [19] is a generalization of Lemma 3.1 in [29].

### Theorem 3.4

*Suppose the following statements hold*:

(i) *D* *is nonempty and bounded*;
(ii) $K_{\infty}\cap\{d\in X:\langle y^{*},d\rangle+J^{\circ}(y,d)+f_{\infty}(d)\leq0, \forall y^{*}\in F(y),y\in K\}=\{0\}$;
(iii) *There exists a bounded set* $C\subset K$ *such that*, *for every* $x\in K\setminus C$, *there exists some* $y\in C$ *satisfying*
  $$ \sup_{y^{*}\in F(y)}\bigl\langle y^{*},x-y\bigr\rangle +J^{\circ}(y,x-y)+f(x)-f(y)>0. $$

*Then* (i)⇒(ii). (ii)⇒(iii) *if* $\operatorname{barr}(K)$ *has nonempty interior*. (iii)⇒(i) *if* *F* *is* $(f,J)$-*pseudomonotone on* *K*.

### Proof

The relationship (i)⇒(ii) can be deduced from Theorem 3.1.

Next, we first prove that (ii)⇒(iii). If (iii) does not hold, then there exists a sequence $\{x_{n}\}\subset K$ such that, for each $n, \|x_{n}\|\geq n$ and $\sup_{y^{*}\in F(y)}\langle y^{*},x_{n}-y\rangle +J^{\circ}(y,x_{n}-y)+f(x_{n})-f(y)\leq0$ for every $y\in K$ with $\|y\|\leq n$. Without loss of generality, we may assume that $d_{n}=x_{n}/\|x_{n}\|$ weakly converges to *d*. Then $d\in K_{\infty}$. By Lemma 2.7, we get $d\neq0$. Let $y\in K$ and $y^{*}\in F(y)$. Then, for all $n>\|y\|$, we have

$$ \begin{aligned} 0&\geq\frac{\langle y^{*},x_{n}-y\rangle+J^{\circ}(y,x_{n}-y)}{ \Vert x_{n} \Vert }+\frac{f( \Vert x_{n} \Vert d_{n})}{ \Vert x_{n} \Vert }- \frac {f(y)}{ \Vert x_{n} \Vert } \\ &\geq\frac{\langle y^{*},x_{n}-y\rangle+J^{\circ}(y,x_{n})-J^{\circ}(y,y)}{ \Vert x_{n} \Vert }+\frac{f( \Vert x_{n} \Vert d_{n})}{ \Vert x_{n} \Vert }-\frac {f(y)}{ \Vert x_{n} \Vert } \\ &=\frac{\langle y^{*},x_{n}-y\rangle-J^{\circ}(y,y)}{ \Vert x_{n} \Vert }+J^{\circ}(y,d_{n})+\frac{f( \Vert x_{n} \Vert d_{n})}{ \Vert x_{n} \Vert }- \frac{f(y)}{ \Vert x_{n} \Vert }. \end{aligned} $$

This together with (2.2) implies that

$$ 0\geq\bigl\langle y^{*},d\bigr\rangle +\liminf_{n\to\infty}J^{\circ}(y,d_{n})+ \liminf_{n\to\infty}\frac{f( \Vert x_{n} \Vert d_{n})}{ \Vert x_{n} \Vert }\geq \bigl\langle y^{*},d\bigr\rangle +J^{\circ}(y,d)+f_{\infty}(d),\quad \forall y^{*}\in F(y), $$

and so

$$ d\in K_{\infty}\cap\bigl\{ d\in X:\bigl\langle y^{*},d\bigr\rangle +J^{\circ}(y,d)+f_{\infty}(d)\leq0, \forall y^{*}\in F(y),y\in K \bigr\} . $$

This implies that

$$ 0\neq d\in K_{\infty}\cap\bigl\{ d\in X:\bigl\langle y^{*},d\bigr\rangle +J^{\circ}(y,d)+f_{\infty}(d)\leq0, \forall y^{*}\in F(y),y\in K \bigr\} , $$

a contradiction to (ii).

It remains to prove that (iii) implies (i) under the assumption that *F* is $(f,J)$-pseudomonotone on *K*. Indeed, let $G:K\to2^{K}$ be a set-valued mapping defined by

$$ G(y):=\Bigl\{ x\in K:\sup_{y^{*}\in F(y)}\bigl\langle y^{*},x-y\bigr\rangle +J^{\circ}(y,x-y)+f(x)-f(y)\leq0\Bigr\} ,\quad \forall y\in K. $$

Firstly, we show that $G(y)$ is a closed subset of *K*. In fact, for any $x_{n}\in G(y)$ with $x_{n}\to x_{0}$, we have

$$ \sup_{y^{*}\in F(y)}\bigl\langle y^{*},x_{n}-y\bigr\rangle +J^{\circ}(y,x_{n}-y)+f(x_{n})-f(y)\leq0. $$

From the lower semicontinuity of *f* and the Lipschitz continuity of $J^{\circ}(\cdot,\cdot)$ in the second variable, it follows that

$$ \begin{aligned} &{ \sup_{y^{*}\in F(y)}}\bigl\langle y^{*},x_{0}-y\bigr\rangle +J^{\circ}(y,x_{0}-y)+f(x_{0})-f(y) \\ &\quad \leq{ \liminf_{n\to\infty}}\Bigl({ \sup_{y^{*}\in F(y)}} \bigl\langle y^{*},x_{n}-y\bigr\rangle \Bigr) +{ \liminf _{n\to\infty}}\bigl(J^{\circ}(y,x_{n}-y)+f(x_{n})-f(y) \bigr)\leq0. \end{aligned} $$

This shows that $x_{0}\in G(y)$ and so $G(y)$ is closed.

Next we prove that $G:K\to K$ is a KKM mapping. If it is not so, then there exist $t_{1},t_{2},\ldots,t_{n}\in[0,1], y_{1},y_{2},\ldots,y_{n}\in K$, and $\bar{y}=t_{1}y_{1}+t_{2}y_{2}+\cdots+t_{n}y_{n}\in\operatorname{conv}\{y_{1},y_{2},\ldots ,y_{n}\}$ such that $\bar{y}\notin\bigcup_{i\in\{1,2,\ldots,n\}}G(y_{i})$. Hence,

$$ \sup_{y^{*}_{i}\in F(y_{i})}\bigl\langle y^{*}_{i},\bar{y}-y_{i}\bigr\rangle +J^{\circ}(y_{i},\bar{y}-y_{i})+f(\bar{y})-f(y_{i})>0,\quad i=1,2,\ldots,n. $$

By the $(f,J)$-pseudomonotonicity of *F*, we get

$$ \sup_{\bar{y}^{*}\in F(\bar{y})}\bigl\langle \bar{y}^{*},\bar{y}-y_{i}\bigr\rangle -J^{\circ}(\bar{y},y_{i}-\bar{y})+f(\bar{y})-f(y_{i})>0,\quad i=1,2,\ldots,n. $$

Since $y\mapsto J^{\circ}(x,y)$ is convex, we deduce

$$ \sum^{n}_{i=1}t_{i}J^{\circ}( \bar{y},y_{i}-\bar{y})\geq J^{\circ}\Biggl(\bar{y},\sum ^{n}_{i=1}t_{i}y_{i}-\bar{y} \Biggr)=J^{\circ}(\bar{y},0)=0, $$

which yields

$$ -\sum^{n}_{i=1}t_{i}J^{\circ}( \bar{y},y_{i}-\bar{y})\leq0. $$

It follows that

$$ \begin{aligned} &f(\bar{y})-{ \sum^{n}_{i=1}}t_{i}f(y_{i}) \\ &\quad \geq{ \sup_{\bar{y}^{*}\in F(\bar{y})}}\Biggl\langle \bar{y}^{*},\bar{y}-{ \sum ^{n}_{i=1}}t_{i}y_{i}\Biggr\rangle -{ \sum^{n}_{i=1}}t_{i}J^{\circ}( \bar{y},y_{i}-\bar{y})+f(\bar{y})-{ \sum^{n}_{i=1}}t_{i}f(y_{i})>0, \end{aligned} $$

and hence

$$ f(\bar{y})>{ \sum^{n}_{i=1}}t_{i}f(y_{i}), $$

which is a contradiction. Therefore, *G* is a KKM mapping.

Assume that *C* is a bounded, closed, and convex (otherwise, we can use the closed convex hull of *C* instead of *C*). Let $\{y_{1},\ldots,y_{m}\}$ be a finite number of points in *K*, and let $M:=\operatorname{conv}(C\cup\{y_{1},\ldots,y_{m}\})$. It is obvious that *M* is weakly compact and convex. Let $G'(y):=G(y)\cap M$ for all $y\in M$. Then $G'(y)$ is a weakly compact and convex subset of *M* and $G'$ is a KKM mapping. We claim that

3.1 $$ \emptyset\neq\bigcap_{y\in M}G'(y) \subset C. $$

Indeed, by Lemma 2.9, the intersection in (3.1) is nonempty. Moreover, if there exists some $x_{0}\in\bigcap_{y\in M}G'(y)$ but $x_{0}\notin C$, then by (iii) we have

$$ \sup_{y^{*}\in F(y)}\bigl\langle y^{*},x_{0}-y\bigr\rangle +J^{\circ}(y,x_{0}-y)+f(x_{0})-f(y)>0 $$

for some $y\in C$. Thus, $x_{0}\notin G(y)$ and so $x_{0}\notin G'(y)$, which is a contradiction to the choice of $x_{0}$.

Let $z\in\bigcap_{y\in M}G'(y)$. Then $z\in C$ by (11) and so $z\in \bigcap^{m}_{i=1}(G(y_{i})\cap C)$. This shows that the collection $\{G(y)\cap C:y\in K\}$ has the finite intersection property. For each $y\in K$, it follows from the weak compactness of $G(y)\cap C$ that $\bigcap_{y\in K}(G(y)\cap C)$ is nonempty, which coincides with the solution set of $\operatorname{GMVHVI}(F,J,K)$. This completes the proof. □

### Corollary 3.5

*Suppose the following statements hold*:

(i) *D* *is nonempty and bounded*;
(ii) $K_{\infty}\cap\{d\in X:\langle y^{*},d\rangle+f_{\infty}(d)\leq 0, \forall y^{*}\in F(y),y\in K\}=\{0\}$;
(iii) *There exists a bounded set* $C\subset K$ *such that*, *for every* $x\in K\setminus C$, *there exists some* $y\in C$ *satisfying*
  $$ \sup_{y^{*}\in F(y)}\bigl\langle y^{*},x-y\bigr\rangle +f(x)-f(y)>0. $$

*Then* (i)⇒(ii). (ii)⇒(iii) *if* $\operatorname{barr}(K)$ *has nonempty interior*. (iii)⇒(i) *if* *F* *is* $(f,J)$-*pseudomonotone on* *K*.

### Remark 3.6

It is known that if $J=0$ then Theorem 3.4 reduces to Theorem 3.2 in Zhong and Huang [19]. Thus, Theorem 3.4 generalizes and extends Theorem 3.2 in Zhong and Huang [19] from $\operatorname{GMMVI}(F,K)$ to $\operatorname{GMVHVI}(F,J,K)$. If $f=0$ additionally, then $f_{\infty}=0$. Consequently, statements (i), (ii), and (iii) in [19, Theorem 3.2] reduce to (i), (ii), and (iii) in [29, Theorem 3.1], respectively. Thus, Zhong and Huang’s Theorem 3.2 in [19] is a generalization of Theorem 3.1 in [29].

## Stability of solution sets

In this section, we will establish the stability of solution sets for the generalized Minty variational-hemivariational inequality $\operatorname{GMVHVI}(F,J,K)$ and the generalized variational-hemivariational inequality $\operatorname{GVHVI}(F,J,K)$ with $(f,J)$-pseudomonotone mappings.

Let $(Z_{1},d_{1})$ and $(Z_{2},d_{2})$ be two metric spaces, $u_{0}\in Z_{1}$ and $v_{0}\in Z_{2}$ be given points. Let $L:Z_{1}\to2^{X}$ be a continuous set-valued mapping with nonempty, closed, and convex values and $\operatorname{int}(\operatorname{barr}L(u_{0}))\neq\emptyset$. Suppose that there exists a neighborhood $U\times V$ of $(u_{0},v_{0})$ such that $M=\bigcup_{u\in U}L(u)$, $F:M\times V\to 2^{X^{*}}$ is a lower semicontinuous set-valued mapping with nonempty values, and let $f:M\subset X\to\mathbf{R}$ be a convex and lower semicontinuous function. Let $J:X\to\mathbf{R}$ be a locally Lipschitz functional such that $J^{\circ}:M\times M\subset X\times X\to\mathbf{R}$ is bi-sequentially weakly lower semicontinuous.

### Theorem 4.1

*If*

4.1 $$ \bigl(L(u_{0})\bigr)_{\infty}\cap\bigl\{ d\in X: \bigl\langle y^{*},d\bigr\rangle +J^{\circ}(y,d)+f_{\infty}(d)\leq0, \forall y^{*}\in F(y,v_{0}), y\in L(u_{0})\bigr\} =\{0\}, $$

*then there exists a neighborhood* $U'\times V'$ *of* $(u_{0},v_{0})$ *with* $U'\times V'\subset U\times V$ *such that*

4.2 $$ \bigl(L(u)\bigr)_{\infty}\cap\bigl\{ d\in X:\bigl\langle y^{*},d\bigr\rangle +J^{\circ}(y,d)+f_{\infty}(d)\leq0, \forall y^{*}\in F(y,v), y\in L(u)\bigr\} =\{0\} $$

*for all* $(u,v)\in U'\times V'$.

### Proof

Assume that the conclusion does not hold. Then there exists a sequence $\{(u_{n},v_{n})\}$ in $Z_{1}\times Z_{2}$ with $(u_{n},v_{n})\to(u_{0},v_{0})$ such that

$$ \bigl(L(u_{n})\bigr)_{\infty}\cap\bigl\{ d\in X:\bigl\langle y^{*},d\bigr\rangle +J^{\circ}(y,d)+f_{\infty}(d)\leq0, \forall y^{*}\in F(y,v_{n}), y\in L(u_{n})\bigr\} \neq\{0\}. $$

Since $f_{\infty}(\lambda x)=\lambda f_{\infty}(x)$ for all $x\in X $ and $\lambda\geq0$, we deduce that

$$ \bigl(L(u_{n})\bigr)_{\infty}\cap\bigl\{ d\in X:\bigl\langle y^{*},d\bigr\rangle +J^{\circ}(y,d)+f_{\infty}(d)\leq0, \forall y^{*}\in F(y,v_{n}), y\in L(u_{n})\bigr\} $$

is a cone. Thus, we can select a sequence $\{d_{n}\}$ such that

$$ d_{n}\in\bigl(L(u_{n})\bigr)_{\infty}\cap\bigl\{ d\in X:\bigl\langle y^{*},d\bigr\rangle +J^{\circ}(y,d)+f_{\infty}(d)\leq0, \forall y^{*}\in F(y,v_{n}), y\in L(u_{n})\bigr\} $$

satisfying $\|d_{n}\|=1$ for every $n=1,2,\ldots$ . Without loss of generality, we can assume that $d_{n}\rightharpoonup d_{0}\ne0$ by Lemma 2.7. By the upper semicontinuity of *L* and Lemma 2.8, we have $(L(u_{n}))_{\infty}\subset(L(u_{0}))_{\infty}$ for large enough *n* and so $d_{n}\in (L(u_{0}))_{\infty}$ for large enough *n*. Since $(L(u_{0}))_{\infty}$ is weakly closed, we have $d_{0}\in(L(u_{0}))_{\infty}$. Take any fixed $y\in L(u_{0})$ and $y^{*}\in F(y,v_{0})$. From the lower semicontinuity of *L*, there exists $y_{n}\in L(u_{n})$ such that $y_{n}\to y$. Hence, $(y_{n},v_{n})\to (y,v_{0})$. By the lower semicontinuity of *F*, there exists $y^{*}_{n}\in F(y_{n},v_{n})$ such that $y^{*}_{n}\to y^{*}$. Since

$$ d_{n}\in\bigl\{ d\in X:\bigl\langle y^{*},d\bigr\rangle +J^{\circ}(y,d)+f_{\infty}(d) \leq0, \forall y^{*}\in F(y,v_{n}), y\in L(u_{n})\bigr\} , $$

we have

$$ \bigl\langle y^{*}_{n},d_{n}\bigr\rangle +J^{\circ}(y_{n},d_{n})+f_{\infty}(d_{n}) \leq0. $$

Combining with $y_{n}\to y, y^{*}_{n}\to y^{*}, d_{n}\rightharpoonup d_{0}$, the bi-sequential weak lower semicontinuity of $J^{\circ}$ and the weak lower semicontinuity of $f_{\infty}$, it follows that $\langle y^{*},d_{0}\rangle+J^{\circ}(y,d_{0})+f_{\infty}(d_{0})\leq0$. Since $y\in L(u_{0})$ and $y^{*}\in F(y,v_{0})$ are arbitrary, from the above discussion, we have

$$ d_{0}\in\bigl\{ d\in X:\bigl\langle y^{*},d\bigr\rangle +J^{\circ}(y,d)+f_{\infty}(d) \leq0, \forall y^{*}\in F(y,v_{0}), y\in L(u_{0})\bigr\} , $$

and so

$$ d_{0}\in\bigl(L(u_{0})\bigr)_{\infty}\cap\bigl\{ d\in X:\bigl\langle y^{*},d\bigr\rangle +J^{\circ}(y,d)+f_{\infty}(d)\leq0, \forall y^{*}\in F(y,v_{0}), y\in L(u_{0})\bigr\} $$

with $d_{0}\neq0$, which contradicts the assumption. This completes the proof. □

### Corollary 4.2

*If*

4.3 $$ \bigl(L(u_{0})\bigr)_{\infty}\cap\bigl\{ d\in X: \bigl\langle y^{*},d\bigr\rangle +f_{\infty}(d)\leq0, \forall y^{*}\in F \bigl(L(u_{0}),v_{0}\bigr)\bigr\} =\{0\}, $$

*then there exists a neighborhood* $U'\times V'$ *of* $(u_{0},v_{0})$ *with* $U'\times V'\subset U\times V$ *such that*

4.4 $$ \bigl(L(u)\bigr)_{\infty}\cap\bigl\{ d\in X:\bigl\langle y^{*},d\bigr\rangle +f_{\infty}(d)\leq0, \forall y^{*}\in F\bigl(L(u),v\bigr) \bigr\} =\{0\} $$

*for all* $(u,v)\in U'\times V'$.

### Proof

Whenever $J=0$, we know that $J^{\circ}=0$ and hence $J^{\circ}$ is bi-sequentially weakly lower semicontinuous. In this case, (4.1) and (4.2) in Theorem 4.1 reduce to (4.3) and (4.4), respectively. Utilizing Theorem 4.1, we immediately deduce Corollary 4.2. □

### Remark 4.3

It is known that if $J=0$ then Theorem 4.1 reduces to Theorem 4.1 in Zhong and Huang [19]. Thus, Theorem 4.1 generalizes and extends Zhong and Huang’s Theorem 4.1 [19] to the case of Clarke’s generalized directional derivative of a locally Lipschitz functional. If $f=0$ additionally, then $f_{\infty}=0$. Thus, (4.3) and (4.4) in Corollary 4.2 reduce to (3.1) and (3.2) in [30, Theorem 3.1], respectively. Therefore, Zhong and Huang’s Theorem 4.1 in [19] is a generalization of Theorem 3.1 in [30].

### Theorem 4.4

*Assume that all the conditions of Theorem *4.1 *are satisfied*. *Suppose that*

(i) *for each* $v\in V$, *the mapping* $x\mapsto F(x,v)$ *is* $(f,J)$-*pseudomonotone on* *M*;
(ii) *the solution set of* $\operatorname{GMVHVI}(F(\cdot,v_{0}),J,L(u_{0}))$ *is nonempty and bounded*.

*Then there exists a neighborhood* $U'\times V'$ *of* $(u_{0},v_{0})$ *with* $U'\times V'\subset U\times V$ *such that*, *for every* $(u,v)\in U'\times V'$, *the solution set of* $\operatorname{GMVHVI}(F(\cdot,v),J,L(u))$ *is nonempty and bounded*. *Moreover*, *if* *f* *is continuous on* $M=\bigcup_{u\in U}L(u)$ *and* $J^{\circ}:M\times(M-M)\to \mathbf{R}$ *is continuous*, *then* *ω*-$\limsup_{(u,v)\to (u_{0},v_{0})}S_{\mathrm{GM}}(u,v) \subset S_{\mathrm{GM}}(u_{0},v_{0})$, *where* $S_{\mathrm{GM}}(u,v)$ *and* $S_{\mathrm {GM}}(u_{0},v_{0})$ *are the solution sets of* $\operatorname{GMVHVI}(F(\cdot,v),J,L(u))$ *and* $\operatorname{GMVHVI}(F(\cdot,v_{0}),J,L(u_{0}))$, *respectively*.

### Proof

By Theorem 3.1, we get

$$ \bigl(L(u_{0})\bigr)_{\infty}\cap\bigl\{ d\in X:\bigl\langle y^{*},d\bigr\rangle +J^{\circ}(y,d)+f_{\infty}(d)\leq0, \forall y^{*}\in F(y,v_{0}), y\in L(u_{0})\bigr\} =\{0\}. $$

It follows from Theorem 4.1 that there exists a neighborhood $U'\times V'$ of $(u_{0},v_{0})$ with $U'\times V'\subset U\times V$ such that

$$ \bigl(L(u)\bigr)_{\infty}\cap\bigl\{ d\in X:\bigl\langle y^{*},d\bigr\rangle +J^{\circ}(y,d)+f_{\infty}(d)\leq0, \forall y^{*}\in F(y,v), y \in L(u)\bigr\} =\{0\} $$

for all $(u,v)\in U'\times V'$. Since *F* is $(f,J)$-pseudomonotone, Theorem 3.4 implies that the solution set of $\operatorname{GMVHVI}(F(\cdot,v),J,L(u))$ is nonempty and bounded for every $(u,v)\in U'\times V'$.

Next, we prove that *ω*-$\limsup_{(u,v)\to(u_{0},v_{0})}S_{\mathrm {GM}}(u,v)\subset S_{\mathrm{GM}}(u_{0},v_{0})$. For $\{(u_{n},v_{n})\}\subset U'\times V'$ with $(u_{n},v_{n})\to(u_{0},v_{0})$, we need to prove that *ω*-$\limsup_{n\to\infty}S_{\mathrm{GM}}(u_{n},v_{n})\subset S_{\mathrm{GM}}(u_{0},v_{0})$. For any $n=0,1,2,\ldots$ , define a function $\varPhi_{n}:X\to\mathbf{R}$ by

$$ \varPhi_{n}(x):=\sup_{y\in L(u_{n}),y^{*}\in F(y,v_{n})}\frac{\langle y^{*},x-y\rangle+J^{\circ}(y,x-y)+f(x)-f(y)}{\varphi(y,y^{*})}, $$

where

$$ \varphi\bigl(y,y^{*}\bigr):=\max\bigl\{ \bigl\Vert y^{*} \bigr\Vert ,1\bigr\} \max\bigl\{ \Vert y \Vert ,1\bigr\} \max\bigl\{ \bigl\vert f(y) \bigr\vert ,1\bigr\} . $$

Let $A_{n}:=\{x\in L(u_{n}):\varPhi_{n}(x)\leq0\}$ for every non-negative integer *n*. By the definition of $\varPhi_{n}$, it is easy to see that $A_{n}=\{x\in L(u_{n}):\varPhi_{n}(x)\leq0\}$ coincides with the solution set $S_{\mathrm{GM}}(u_{n},v_{n})$ of $\operatorname{GMVHVI}(F(\cdot,v),J,L(u))$ for all $n=0,1,2,\ldots$ . Thus, $A_{n}$ is nonempty and bounded by condition (ii) for every non-negative integer *n*. From the above discussion, we need only to prove that *ω*-$\limsup_{n\to\infty}A_{n}\subset A_{0}$. Let $x\in\omega $-$\limsup_{n\to\infty}A_{n}$. Then there exists a sequence $\{x_{n_{j}}\}$ with each $x_{n_{j}}\in A_{n_{j}}$ such that $x_{n_{j}}$ weakly converges to *x*. We claim that there exists $z_{n_{j}}\in L(u_{0})$ such that $\lim_{j\to\infty}\|x_{n_{j}}-z_{n_{j}}\|=0$. Indeed, if the claim does not hold, then there exist a subsequence $\{x_{n_{j_{k}}}\}$ of $\{ x_{n_{j}}\}$ and some $\varepsilon_{0}>0$ such that

$$ d\bigl(x_{n_{j_{k}}},L(u_{0})\bigr)\geq\varepsilon_{0},\quad k=1,2,\ldots. $$

This implies that $x_{n_{j_{k}}}\notin L(u_{0})+\varepsilon_{0}B(0,1)$ and so $L(u_{n_{j_{k}}})\not\subset L(u_{0})+\varepsilon_{0}B(0,1)$, which contradicts the upper semicontinuity of $L(\cdot)$. Moreover, we obtain $x\in L(u_{0})$ as $L(u_{0})$ is a closed and convex subset of *X* and hence weakly closed. Next we prove that $\varPhi_{0}(x)\leq0$ and hence $x\in A_{0}$. In fact, for any fixed $y\in L(u_{0})$ and $y^{*}\in F(y,v_{0})$, since *L* is lower semicontinuous and $u_{n}\to u_{0}$, we know that there exists $y_{n}\in L(u_{n})$ for every $n=1,2,\ldots$ such that $\lim_{n\to\infty}y_{n}=y$. Since *F* is lower semicontinuous, it follows that there exists a sequence of elements $y^{*}_{n}\in F(y_{n},v_{n})$ such that $y^{*}_{n}\to y^{*}$. Now $x_{n_{j}}\in A_{n_{j}}$ implies that $\varPhi_{n_{j}}(x_{n_{j}})\leq0$ and so

$$ \frac{\langle y^{*}_{n_{j}},x_{n_{j}}-y_{n_{j}}\rangle+J^{\circ}(y_{n_{j}},x_{n_{j}}-y_{n_{j}})+f(x_{n_{j}})-f(y_{n_{j}})}{\varphi (y_{n_{j}},y^{*}_{n_{j}})}\leq0. $$

Since *f* is continuous on $M=\bigcup_{u\in U}L(u)$ and $J^{\circ}:M\times (M-M)\to\mathbf{R}$ is also continuous, letting $j\to\infty$, we have

$$ \frac{\langle y^{*},x-y\rangle+J^{\circ}(y,x-y)+f(x)-f(y)}{\varphi (y,y^{*})}\leq0. $$

Since $y\in L(u_{0})$ and $y^{*}\in F(y,v_{0})$ are arbitrary, we know that $\varPhi_{0}(x)\leq0$ and hence $x\in A_{0}$. This completes the proof. □

### Corollary 4.5

*Assume that all the conditions of Corollary *4.2 *are satisfied*. *Suppose that*

(i) *for each* $v\in V$, *the mapping* $x\mapsto F(x,v)$ *is* *f*-*pseudomonotone on* *M*;
(ii) *the solution set of* $\operatorname{GMMVI}(F(\cdot,v_{0}),L(u_{0}))$ *is nonempty and bounded*.

*Then there exists a neighborhood* $U'\times V'$ *of* $(u_{0},v_{0})$ *with* $U'\times V'\subset U\times V$ *such that*, *for every* $(u,v)\in U'\times V'$, *the solution set of* $\operatorname{GMMVI}(F(\cdot,v),L(u))$ *is nonempty and bounded*. *Moreover*, *if* *f* *is continuous on* $M=\bigcup_{u\in U}L(u)$, *then* *ω*-$\limsup_{(u,v)\to (u_{0},v_{0})}S_{M}(u,v)\subset S_{M}(u_{0},v_{0})$, *where* $S_{M}(u,v)$ *and* $S_{M}(u_{0},v_{0})$ *are the solution sets of* $\operatorname{GMMVI}(F(\cdot,v),L(u))$ *and* $\operatorname{GMMVI}(F(\cdot ,v_{0}),L(u_{0}))$, *respectively*.

### Proof

Whenever $J=0$, we know that $J^{\circ}=0$, $\operatorname{GMVHVI}(F(\cdot ,v),J,L(u))$ (resp., $\operatorname{GMVHVI}(F(\cdot, v_{0}),J,L(u_{0}))$) reduces to $\operatorname{GMMVI}(F(\cdot,v),L(u))$ (resp., $\operatorname{GMMVI}(F(\cdot,v_{0}),L(u_{0}))$), $S_{\mathrm{GM}}(u,v)$ (resp., $S_{\mathrm{GM}}(u_{0},v_{0})$) reduces to $S_{M}(u,v)$ (resp., $S_{M}(u_{0},v_{0})$), and the $(f,J)$-pseudomonotonicity of *F* in the first variable reduces to the *f*-pseudomonotonicity of *F* in the first variable. Utilizing Theorem 4.9, we immediately deduce Corollary 4.5. □

### Remark 4.6

It is known that if $J=0$ then Theorem 4.4 reduces to Theorem 4.2 in Zhong and Huang [19]. Thus, Theorem 4.4 generalizes and extends Theorem 4.2 in Zhong and Huang [19] from the generalized Minty mixed variational inequality to the generalized Minty variational-hemivariational inequality. If $f=0$ additionally, then $f_{\infty}=0$, and so the generalized Minty mixed variational inequality $\operatorname{GMMVI}(F,K)$ reduces to the generalized Minty variational inequality. Hence, Zhong and Huang’s Theorem 4.2 [19] generalizes [30, Theorem 3.2] from the generalized Minty variational inequality to the generalized Minty mixed variational inequality. In addition, for the case of $J=f=0$, He [29] obtained the corresponding result of Zhong and Huang’s Theorem 4.2 [19] when either the mapping or the constraint set is perturbed (see Theorems 4.1 and 4.4 of [29]). Therefore, Zhong and Huang’s Theorem 4.2 [19] is a generalization of Theorems 4.1 and 4.4 in [29].

In the following, as an application of Theorem 4.4, we will consider the stability behavior for the following generalized variational-hemivariational inequality, denoted by $\operatorname{GVHVI}(F,J,K)$, which is to find $x\in K$ and $x^{*}\in F(x)$ such that

4.5 $$ \operatorname{GVHVI}(F,J,K): \bigl\langle x^{*},y-x\bigr\rangle +J^{\circ}(x,y-x)+f(y)-f(x) \geq 0, \quad \forall y\in K. $$

If $J=0$, then $\operatorname{GVHVI}(F,J,K)$ reduces to the generalized mixed variational inequality, which is to find $x\in K$ and $x^{*}\in F(x)$ such that

4.6 $$ \operatorname{GMVI}(F,K): \bigl\langle x^{*},y-x\bigr\rangle +f(y)-f(x)\geq0,\quad \forall y\in K. $$

If *F* is single-valued, then (4.5) reduces to (1.1). Furthermore, if $f=0$, then (4.6) reduces to the following generalized variational inequality of finding $x\in K$ and $x^{*}\in F(x)$ such that

4.7 $$ \operatorname{GVI}(F,K): \bigl\langle x^{*},y-x\bigr\rangle \geq0, \quad\forall y \in K. $$

Next we consider the parametric generalized variational-hemivariational inequality, denoted by $\operatorname{GVHVI}(F(\cdot,v),J,L(u))$, which is to find $x\in L(u)$ and $x^{*}\in F(x,v)$ such that

4.8 $$ \operatorname{GVHVI}\bigl(F(\cdot,v),J,L(u)\bigr): \bigl\langle x^{*},y-x \bigr\rangle +J^{\circ}(x,y-x)+f(y)-f(x)\geq0, \quad\forall y\in L(u). $$

In particular, if $J=0$, then (4.8) reduces to the following parametric generalized mixed variational inequality, which is to find $x\in L(u)$ and $x^{*}\in F(x,v)$ such that

4.9 $$ \operatorname{GMVI}\bigl(F(\cdot,v),L(u)\bigr): \bigl\langle x^{*},y-x\bigr\rangle +f(y)-f(x)\geq0, \quad\forall y\in L(u). $$

The following lemma shows that $\operatorname{GVHVI}(F,J,K)$ is closely related to its generalized Minty variational-hemivariational inequality.

### Lemma 4.7

(i) *If* *F* *is* $(f,J)$-*pseudomonotone on* *K*, *then every solution of* $\operatorname{GVHVI}(F,J,K)$ *solves* $\operatorname{GMVHVI}(F,J,K)$. (ii) *If* *F* *is upper hemicontinuous on* *K* *with nonempty values*, *then every solution of* $\operatorname{GMVHVI}(F,J,K)$ *solves* $\operatorname{GVHVI}(F,J,K)$.

### Proof

(i) The conclusion is obvious. Now we prove (ii). Suppose that *x* is a solution of $\operatorname{GMVHVI}(F,J,K)$, but it is not a solution of $\operatorname{GVHVI}(F,J,K)$. Then there exists some $y\in K$ such that

$$ \bigl\langle x^{*},y-x\bigr\rangle +J^{\circ}(x,y-x)+f(y)-f(x)< 0, \quad \forall x^{*} \in F(x). $$

Since the set $\{x^{*}\in X^{*}:\langle x^{*},y-x\rangle+J^{\circ}(x,y-x)+f(y)-f(x)<0\}$ is a weakly^∗^ open neighborhood of $F(x)$ and *F* is upper hemicontinuous, setting $x_{t}=ty+(1-t)x$ for $t>0$ small enough, we deduce from the positive homogeneousness of $J^{\circ}$ in the second variable

$$ \bigl\langle x^{*}_{t},y-x\bigr\rangle +J^{\circ}(x_{t},y-x)+f(y)-f(x)< 0. $$

It follows that, for any $t>0$,

4.10 $$ \bigl\langle x^{*}_{t},t(y-x)\bigr\rangle +J^{\circ}\bigl(x_{t},t(y-x)\bigr)+t\bigl(f(y)-f(x)\bigr)< 0. $$

By the convexity of *f*, we have

$$ f(x_{t})=f\bigl(ty+(1-t)x\bigr)\leq tf(y)+(1-t)f(x) $$

and so $f(x_{t})-f(x)\leq t(f(y)-f(x))$. Utilizing (4.10) and the subadditivity of $J^{\circ}$ in the second variable, we obtain that

$$ \begin{aligned} &\bigl\langle x^{*}_{t},x_{t}-x \bigr\rangle -J^{\circ}(x_{t},x-x_{t})+f(x_{t})-f(x) \\ &\quad\leq\bigl\langle x^{*}_{t},x_{t}-x\bigr\rangle +J^{\circ}(x_{t},x_{t}-x)+f(x_{t})-f(x) \\ &\quad\leq\bigl\langle x^{*}_{t},x_{t}-x\bigr\rangle +J^{\circ}(x_{t},x_{t}-x)+t\bigl(f(y)-f(x) \bigr)< 0, \end{aligned} $$

which immediately leads to

$$ \bigl\langle x^{*}_{t},x-x_{t}\bigr\rangle +J^{\circ}(x_{t},x-x_{t})+f(x)-f(x_{t})>0. $$

This contradicts the fact that *x* is a solution of $\operatorname{GMVHVI}(F,J,K)$. Hence, the conclusion of (ii) holds. This completes the proof. □

### Corollary 4.8

(i) *If* *F* *is* *f*-*pseudomonotone on* *K*, *then every solution of* $\operatorname{GMVI}(F,K)$ *solves* $\operatorname{GMMVI}(F,K)$. (ii) *If* *F* *is upper hemicontinuous on* *K* *with nonempty values*, *then every solution of* $\operatorname{GMMVI}(F,K)$ *solves* $\operatorname{GMVI}(F,K)$.

### Proof

Whenever $J=0$, we know that $J^{\circ}=0$, $\operatorname{GMVHVI}(F,J,K)$ (resp., $\operatorname{GVHVI}(F,J,K)$) reduces to $\operatorname{GMMVI}(F,K)$ (resp., $\operatorname{GMVI}(F,K)$), and the $(f,J)$-pseudomonotonicity of *F* reduces to the *f*-pseudomonotonicity of *F*. Utilizing Lemma 4.7, we immediately deduce Corollary 4.8. □

### Lemma 4.9

*Let* *K* *be a nonempty*, *closed*, *and convex subset in a reflexive Banach space* *X*, $f:K\subset X\to\mathbf{R}$ *be a convex and lower semicontinuous function*, *and* $J:X\to\mathbf{R}$ *be a locally Lipschitz functional*. *Suppose that* *F* *is upper hemicontinuous and* $(f,J)$-*pseudomonotone on* *K* *with nonempty values*. *Consider the following statements*:

(i) *the solution set of* $\operatorname{GVHVI}(F,J,K)$ *is nonempty and bounded*;
(ii) *the solution set of* $\operatorname{GMVHVI}(F,J,K)$ *is nonempty and bounded*;
(iii) $K_{\infty}\cap\{d\in X:\langle y^{*},d\rangle+J^{\circ}(y,d)+f_{\infty}(d)\leq0, \forall y^{*}\in F(y), y\in K\}=\{0\}$.

*Then* (i)⇔(ii) *and* (ii)⇒(iii); *moreover*, *if* $\operatorname{int}(\operatorname{barr}(K))\neq\emptyset$, *then* (iii)⇒(ii) *and hence they all are equivalent*.

### Proof

Under the assumptions of *F*, the equivalence of (i) and (ii) is stated in Lemma 4.7. Then the conclusion follows from Theorem 3.4. □

### Corollary 4.10

*Let* *K* *be a nonempty*, *closed*, *and convex subset in a reflexive Banach space* *X* *and* $f:K\subset X\to\mathbf{R}$ *be a convex and lower semicontinuous function*. *Suppose that* *F* *is upper hemicontinuous and* *f*-*pseudomonotone on* *K* *with nonempty values*. *Consider the following statements*:

(i) *the solution set of* $\operatorname{GMVI}(F,K)$ *is nonempty and bounded*;
(ii) *the solution set of* $\operatorname{GMMVI}(F,J,K)$ *is nonempty and bounded*;
(iii) $K_{\infty}\cap\{d\in X:\langle y^{*},d\rangle+f_{\infty}(d)\leq 0, \forall y^{*}\in F(y), y\in K\}=\{0\}$.

*Then* (i)⇔(ii) *and* (ii)⇒(iii); *moreover*, *if* $\operatorname{int}(\operatorname{barr}(K))\neq\emptyset$, *then* (iii)⇒(ii) *and hence they all are equivalent*.

### Proof

Whenever $J=0$, we know that $J^{\circ}=0$, the $(f,J)$-pseudomonotonicity of *F* reduces to the *f*-pseudomonotonicity of *F*, and statements (i), (ii), and (iii) in Lemma 4.9 reduce to (i), (ii), and (iii) in Corollary 4.10. Utilizing Lemma 4.9, we deduce the desired result. □

### Remark 4.11

It is known that if $J=0$ then Lemmas 4.7 and 4.9 reduce to Lemmas 4.1 and 4.2 in [19], respectively. Thus, Lemmas 4.7 and 4.9 generalize and extend Lemmas 4.1 and 4.2 in [19] from the generalized mixed variational inequality to the generalized variational-hemivariational inequality. If $f=0$ additionally, then Lemma 4.2 in [19] reduces to Theorem 3.2 of [29]. Therefore, Lemma 4.2 in [19] generalizes Theorem 3.2 of [29] from the generalized variational inequality to the generalized mixed variational inequality.

From Theorem 4.4 and Lemma 4.9, we can easily establish the following stability result for the generalized variational-hemivariational inequality.

### Theorem 4.12

*Assume that all the conditions of Theorem *4.1 *are satisfied*. *Suppose that*

(i) *for each* $v\in V$, *the mapping* $x\mapsto F(x,v)$ *is upper hemicontinuous and* $(f,J)$-*pseudomonotone on* *M*;
(ii) *the solution set of* $\operatorname{GVHVI}(F(\cdot,v_{0}),J,L(u_{0}))$ *is nonempty and bounded*.

*Then there exists a neighborhood* $U'\times V'$ *of* $(u_{0},v_{0})$ *with* $U'\times V'\subset U\times V$ *such that*, *for every* $(u,v)\in U'\times V'$, *the solution set of* $\operatorname{GVHVI}(F(\cdot,v),J,L(u))$ *is nonempty and bounded*. *Moreover*, *if* *f* *is continuous on* $M=\bigcup_{u\in U}L(u)$ *and* $J^{\circ}:M\times(M-M)\to\mathbf{R}$ *is continuous*, *then* *ω*-$\limsup_{(u,v)\to(u_{0},v_{0})}S_{G}(u,v) \subset S_{G}(u_{0},v_{0})$, *where* $S_{G}(u,v)$ *and* $S_{G}(u_{0},v_{0})$ *are the solution sets of* $\operatorname{GVHVI}(F(\cdot,v),J,L(u))$ *and* $\operatorname{GVHVI}(F(\cdot,v_{0}),J,L(u_{0}))$, *respectively*.

### Proof

Since *F* is upper hemicontinuous with nonempty values and $(f,J)$-pseudomonotone on *M*, it follows from Lemma 4.9 that the solution set of $\operatorname{GMVHVI}(F(\cdot,v),J,L(u))$ coincides with that of $\operatorname{GVHVI}(F(\cdot,v),J,L(u))$, and so the result follows directly from Theorem 4.4. This completes the proof. □
